# Comorbidities of overweight and obesity associated risk factor in Saudi Arabia: a population-based analysis

**DOI:** 10.1080/16549716.2025.2477387

**Published:** 2025-04-09

**Authors:** Arwa M. Alshangiti, Mohammed S. Aldossary, Abdulaziz I. Abou-Hussein, Wejdan J. Aloufi, Mervat M. El Dalatony, Shaker A. Alomary

**Affiliations:** a General Directorate of Statistics and Information, Ministry of Health, Riyadh, Saudi Arabia; b Department of Statistics and Operation Research, College of Science, King Saud University, Riyadh, Saudi Arabia; c General Directorate of Research and Studies, Ministry of Health, Riyadh, Saudi Arabia; d Public Health & Community Medicine Department, Faculty of Medicine, Menoufia University, Menoufia, Egypt; e General Directorate of Health Programs and Chronic Diseases, Ministry of Health, Riyadh, Saudi Arabia

**Keywords:** Obesity, overweight, prevalence, risk factors, Saudi Arabia

## Abstract

**Background:**

Obesity is a significant public health challenge in the Kingdom of Saudi Arabia (KSA), with profound impacts on individual well-being and the healthcare system. Recent epidemiological studies have reported variable trends in obesity prevalence within the country. This population-based study aimed to estimate the prevalence, identify behavioral risk factors, and assess comorbidities associated with overweight and obesity using a nationally representative sample in KSA. Findings will inform targeted public health policies, optimize healthcare resource allocation, and support Saudi Vision 2030 goals by promoting healthier lifestyles and reducing chronic diseases.

**Methods:**

This study analyzed data from the 2019 Kingdom of Saudi Arabia World Health Survey (KSAWHS), implemented by the Ministry of Health using a nationally representative sample. A stratified, three-stage sampling design based on the 2010 Census was used to select 10,000 households from 13 administrative regions. Data collection included socio-demographic, anthropometric measurements and medical information from consenting individuals. Continuous variables were summarized as mean ± SD, and univariate analysis was performed using one-way ANOVA and Chi-square tests. Logistic regression identified predictors of obesity and overweight, reporting odds ratios (OR) with 95% confidence intervals (CI). Analyses were conducted in SPSS (v29).

**Results:**

A total of 7930 adults across different regions in KSA were included in this study. The overall prevalence of obesity and overweight was 20.3% and 38.7%, respectively. Overweight was significantly more prevalent in males (44.4%) than females (35.6%), whereas obesity was more common in females (22.1%) compared to males (18.6%) (*p* < 0.001). Regional differences were observed with the West region reporting the lowest prevalence of obesity (16.5%) and overweight (37.3%) (*p* < 0.001). Married individuals exhibited a significantly higher prevalence of both obesity and overweight (*p* < 0.001).

**Conclusion:**

The burden of obesity and overweight in KSA is still alarming due to the associated risk of metabolic, cardiovascular, and psychological disorders, affecting both patients and the healthcare system. Urgent interventions, including targeted public health campaigns, lifestyle modifications, and policy-driven strategies, are essential to curb obesity trends and promote long-term health improvements.

## Paper Context

- **Main findings:** The prevalence of obesity and overweight in KSA was 20.3% and 38.7%, respectively, indicating a substantial public health burden. Obesity was significantly associated with metabolic, cardiovascular, and psychological disorders. Key behavioral risk factors included physical inactivity, poor dietary habits, and smoking.
- **Added knowledge:** Provides updated, nationally representative data on obesity trends and risk factors in KSA. Highlights the role of lifestyle behaviors in obesity prevalence and associated comorbidities. Identifies critical targets for intervention, emphasizing the need for evidence-based health strategies.
- **Global health impact for policy and action:** Supports the development of targeted public health policies to combat obesity-related diseases. Aligns with global efforts to reduce non-communicable diseases (NCDs) through preventive strategies. Reinforces the need for multi-sectoral approaches, including healthcare, education, and urban planning, to promote healthier lifestyles and reduce obesity rates.

## Introduction

Obesity has emerged as a significant global public health challenge, with nearly 650 million adults worldwide currently living with obesity, according to the World Health Organization (WHO) [1]. Projections suggest that by 2035, more than half of the global adult population will be obese or overweight [2]. This alarming increase is driven by the interplay of behavioral, environmental, and socioeconomic determinants [3,4]. Rapid urbanization and technological advancements have led to reduced physical activity and a growing reliance on energy-dense, nutrient-poor diets [5]. Sociocultural norms in many regions further exacerbate this issue by encouraging sedentary lifestyles and promoting calorically dense traditional foods [6]. Additionally, genetic and epigenetic factors have been shown to increase susceptibility to weight gain and limit the effectiveness of weight management strategies [7].

The burden of obesity and overweight is multifaceted, encompassing clinical, humanistic, and economic consequences. Clinically, both conditions are established primary risk factors for a wide range of non-communicable diseases (NCDs), including cardiovascular disease, type 2 diabetes mellitus (T2DM), hypertension, fatty liver disease, osteoarthritis, and sleep apnea [8,9]. Humanistic impacts include diminished quality of life, psychological distress, and social stigmatization [10]. Economically, the direct and indirect costs associated with obesity are substantial, with an estimated economic impact of USD 4.32 trillion by 2035, equivalent to nearly 3% of the global economy [11]. Addressing this growing burden requires an in-depth understanding of the prevalence and risk factors of obesity and overweight to inform evidence-based prevention and intervention strategies.

The Middle East, including the Kingdom of Saudi Arabia (KSA), has witnessed a significant increase in the prevalence of obesity and overweight over recent decades. The Global Burden of Disease (GBD) study reported a 7% increase in obesity prevalence in the region between 1980 and 2015, reaching an overall prevalence of 21% [12]. Among Gulf Cooperation Council (GCC) countries, the prevalence is notably higher. For example, 32.3% of adults in the United Arab Emirates (UAE) are obese, while 43% are overweight [13]. In KSA, estimates for obesity prevalence among adults have varied, ranging from 28.7% to 35.6% in earlier studies [14], aligning with the 2016 WHO report showing 68.2% and 33.7% prevalence for overweight and obesity, respectively [15]. However, recent surveys indicate a decline in obesity prevalence to 23–24.7%, potentially reflecting the success of government initiatives, public health campaigns, and increased physical activity among the population [16,17].

Despite these findings, the need for a comprehensive, nationally representative study on obesity and overweight prevalence, risk factors, and comorbidities in KSA remains critical. Limited documentation exists regarding behavioral and socioeconomic risk factors for these conditions [18]. This study aims to address this gap by analyzing a nationally representative sample to identify the prevalence, risk factors, and associated comorbidities of overweight and obesity in KSA. These findings will provide a critical baseline as the Saudi Ministry of Health (MoH) prepares for its national health survey in 2024, in alignment with WHO recommendations.

## Materials and methods

### Study design and ethical approval

This secondary analysis was conducted in accordance with the STROBE statement guidelines [19]. Data for the analysis were derived from the 2019 Kingdom of Saudi Arabia World Health Survey (KSAWHS 2019) [20]. The original survey adhered to the principles outlined in the latest version of the Declaration of Helsinki and complied with local regulatory laws. Ethical approval was obtained from the Institutional Review Board (IRB) of the Saudi Ministry of Health (IRB No. 23-45M). Written informed consent was secured from all participants during the data collection process for the registry.

### Study design and data source

The KSAWHS 2019 was a population-based survey designed to gather comprehensive health-related data at national and subnational levels in Saudi Arabia [20]. Conducted by the Ministry of Health, the survey aimed to produce reliable, up-to-date estimates of priority health indicators to inform healthcare policy and intervention strategies.

A stratified three-stage sampling design was used to ensure national representativeness. The Master Sample Frame (MSF), based on the 2010 Population and Housing Census conducted by the General Authority of Statistics (GASTAT), was employed to select participants [20]. The sampling framework divided the Kingdom into administrative regions and subregions, further subdividing them into Quarters and census enumeration areas (EAs), which served as primary sampling units (PSUs). A total of 10,000 households were sampled proportionally to population size across all regions.

The survey was administered to a consenting individual, randomly selected from the household roster. Data collected included sociodemographic characteristics, behavioral factors, and anthropometric measurements.

For this analysis, data were restricted to adults aged ≥18 years with complete physical measurements necessary to calculate the body mass index (BMI). After applying these inclusion criteria, the final analytic sample consisted of 7,930 individuals.

### Variables and definitions

Variables were selected based on the WHO STEPwise approach to NCD examinations [21]. Sociodemographic data included age, sex, marital status, and occupational status. Behavioral risk factors, such as physical inactivity, smoking (tobacco use in the past 30 days), and dietary habits (fruit and vegetable intake), were also retrieved. Comorbidities were self-reported, with Type 2 Diabetes Mellitus (T2DM), dyslipidemia, and cardiovascular diseases identified based on participant reports. Hypertension was defined as either self-reported high blood pressure or a new diagnosis based on measured systolic blood pressure (≥140 mmHg) or diastolic blood pressure (≥90 mmHg). Blood pressure was recorded using an electronic monitor, with three readings taken 10 min apart, and the average used for analysis. Anthropometric measurements followed standardized protocols, with height measured using a calibrated measuring tape or stadiometer, ensuring participants removed footwear and heavy clothing. Weight was recorded to the nearest 0.1 kg, and BMI was calculated as weight (kg) divided by height squared (m^2^). BMI classifications followed WHO guidelines: underweight (<18.5), normal weight (18.5–24.9), overweight (25–29.9), and obese (≥30) [21].

Data analysis was conducted using IBM SPSS Statistics for Windows (Version 29.0). Continuous variables were summarized as mean ± standard deviation (SD), while categorical variables were presented as frequencies and percentages. Associations between BMI categories and continuous variables were evaluated using one-way analysis of variance (ANOVA), while associations with categorical variables were assessed using the Chi-square test.

To identify predictors of overweight and obesity, multivariable logistic regression analysis was performed. Odds ratios (ORs) with 95% confidence intervals (95% CI) were calculated. Statistical significance was set at a p-value of <0.05.

## Results

Out of 10,000 questionnaires, this study population comprised 7,930 adults, with males representing the majority (54.5%). The geographical distribution of participants was comparable across regions: 22.6% from the South, 22.5% from the Central region, 21.6% from the North, 21.0% from the West, and 12.2% from the East.

**Figure 1.**
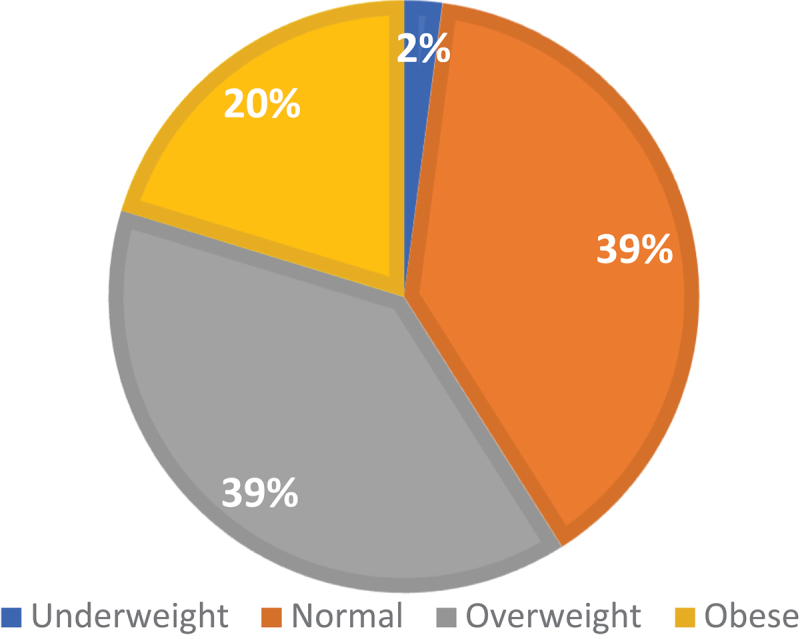
Prevalence of obesity and overweight in KSA.

The overall prevalence of overweight and obesity was 38.7% and 20.3%, respectively (Figure 1). Significant differences in age were observed across BMI categories, with the mean age progressively increasing from 33.08 ± 11.69 years in the normal BMI group to 41.44 ± 13.18 years in the obese category (*p* < 0.001).

Sex-based differences in BMI categories were notable. Overweight was more prevalent in males (44.4%) than females (35.6%), while obesity was higher among females (22.1%) compared to males (18.6%) (*p* < 0.001). Regional variations in BMI were significant, with the West region reporting the lowest prevalence of obesity (16.5%) and overweight (37.3%) compared to other regions (*p* < 0.001).

Marital status also influenced BMI, with married individuals having significantly higher rates of both obesity and overweight compared to unmarried individuals (*p* < 0.001) (Table 1).

**Table 1.** General characteristics among BMI categories of survey responders.

Variables	Normal		Overweight		Obese		*p-value*
**Age (years)**, mean ± SD	33.08 ± 11.69		37.49 ± 12.37		41.44 ± 13.18		*<0.001*
**Gender n (%)**							*<0.001*
Male	1599	37.0%	1917	44.4%	804	18.6%	
Female	1526	42.3%	1285	35.6%	799	22.1%	
**Total**	3125	39.41%	3202	40.4%	1603	20.2%	
**Region n (%)**							*<0.001*
West	771	46.2%	622	37.3%	275	16.5%	
East	318	32.9%	379	39.2%	270	27.9%	
South	681	37.9%	718	40.0%	396	22.1%	
North	665	38.8%	766	44.7%	283	16.5%	
Central	690	38.6%	717	40.1%	379	21.2%	
**Total**	3125	39.41%	3202	40.4%	1603	20.2%	
**Marital status n (%)**							*<0.001*
Single	846	55.0%	518	33.7%	175	11.4%	
Married	2120	36.2%	2465	42.1%	1273	21.7%	
Unmarried (divorced/widowed)	159	29.8%	219	41.1%	155	29.1%	
**Total**	3125	39.41%	3202	40.4%	1603	20.2%	
**Current work status n (%)**							*<0.001*
Working	1557	36.5%	1888	44.3%	816	19.2%	
Not-working	1568	42.7%	1314	35.8%	787	21.4%	
**Total**	3125	39.41%	3202	40.4%	1603	20.2%	

Analysis of behavioral risk factors revealed significant differences in BMI categories regarding tobacco consumption. Individuals who did not consume tobacco demonstrated a lower prevalence of both obesity (19.8% vs. 22.9%) and overweight (39.6% vs. 45.2%) compared to those who were current tobacco users (*p* < 0.001).

In contrast, daily consumption of vegetables and fruits was not significantly associated with either obesity or overweight. The prevalence of obesity and overweight did not differ significantly between individuals consuming varying amounts of daily vegetable servings (*p* = 0.463) or daily fruit servings (*p* = 0.584). A standard vegetable serving was defined as 75 g, while a standard fruit serving was 150 g (Table 2).

**Table 2.** Distribution of some behavioral risk factors by BMI categories among the Saudi responders.

Variables	Normal n(%)		Overweight n(%)		Obese n(%)		*p-value*
**Current tobacco consumption**							
No	2715	40.6%	2643	39.6%	1324	19.8%	*<0.001*
Yes	353	31.9%	500	45.2%	253	22.9%	
**Total**	3068	39.39%	3143	40.4%	1577	20.2%	
**How many servings of vegetables do you have daily?**							
<5 servings/day	2902	39.3%	2991	40.5%	1499	20.3%	*0.463*
≥5 servings/day	38	33.6%	49	43.4%	26	23.0%	
**Total**	2940	39.2%	3040	40.5%	1525	20.3%	
**How many servings of fruits do you have daily?**							
<5 servings/day	2226	38.8%	2340	40.8%	1176	20.5%	*0.584*
≥5 servings/day	39	43.8%	32	36.0%	18	20.2%	
**Total**	2265	38.84%	2372	40.68%	1194	20.48%	

The multivariate logistic regression analysis identified several significant predictors of overweight and obesity. Age was a strong determinant, with individuals in all age groups above 18–29 years (reference group) exhibiting significantly higher odds of being overweight or obese. The highest odds were observed among those aged 50–59 years (OR: 2.949, *p* = 0.001). Marital status and employment status also influenced the odds of being overweight or obese. Married individuals had 33% higher odds (OR: 1.333, *p* = 0.001), while non-working individuals had 19% higher odds (OR: 1.194, *p* = 0.011) compared to their counterparts. Behavioral factors, such as tobacco use, were associated with increased risk; tobacco users had 36% higher odds (OR: 1.355, *p* = 0.001) of being overweight or obese compared to non-users. Among reported comorbidities, dyslipidemia (OR: 2.710, *p* = 0.001) and hypertension (OR: 1.961, *p* = 0.001) were significant predictors of overweight and obesity (Table 3).

**Table 3.** Logistic regression analysis of the risk factors associated with overweight and obesity.

Variables	Adjusted OR (95% CI)	P value*
**Gender**		
Male	Reference	
Female	0.922(0.807 to 1.053)	0.229
**Marital status**		
Single	Reference	
Married	1.333(1.149 to 1.546)	**0.001**
Unmarried (divorced/widowed)	1.196(0.916 to 1.563)	0.189
**Current work status**		
Working	Reference	
Not-working	1.194(1.042 to 1.368)	**0.011**
**Diabetes mellitus**		
No	Reference	
Yes	1.172(0.920 to 1.492)	0.199
**Tobacco**		
No	Reference	
Yes	1.355(1.147 to 1.599)	**0.001**
**Dyslipidemia**		
No	Reference	
Yes	2.710(2.031 to 3.616)	**0.001**
**Hypertension**		
No	Reference	
Yes	1.961(1.487 to 2.586)	**0.001**
**Coronary heart disease**		
No	Reference	
Yes	3.139(0.929 to 10.607)	0.066
**Age group (years)**		
18–29	Reference	
30–39	1.785(1.564 to 2.036)	**0.001**
40–49	2.197(1.836 to 2.629)	**0.001**
50–59	2.949(2.279 to 3.816)	**0.001**
60+	1.658(1.197 to 2.297)	0.002

*p < 0.05 indicates a statistically significant difference.

Table 4 highlights the distribution of comorbidities across BMI categories. The prevalence of diabetes mellitus was significantly higher among overweight (39.5%) and obese (39.2%) individuals compared to those with a normal BMI (21.3%; *p* < 0.001). Similarly, dyslipidemia prevalence increased progressively with BMI, being highest among obese individuals (52.3%) and overweight individuals (35.1%; *p* < 0.001). A comparable pattern was observed for hypertension, with 45.1% of obese individuals affected, compared to 39.3% of overweight individuals and 15.6% of those with a normal BMI (*p* < 0.001).

**Table 4.** Distribution of some medical conditions among BMI categories among the Saudi responders.

Variables	Normal N (%)		Overweight N (%)		Obese N (%)		*p-value*
**Diabetes mellitus**							*<0.001*
No	2990	41.0%	2952	40.5%	1355	18.6%	
Yes	135	21.3%	250	39.5%	248	39.2%	
**Total**	3125	39.41%	3202	40.38%	1603	20.21%	
**Dyslipidemia**							*<0.001*
No	3055	41.4%	3006	40.8%	1311	17.8%	
Yes	70	12.5%	196	35.1%	292	52.3%	
**Total**	3125	39.41%	3202	40.38%	1603	20.21%	
**Hypertension**							*<0.001*
No	3029	41.4%	2960	40.5%	1325	18.1%	
Yes	96	15.6%	242	39.3%	278	45.1%	
**Total**	3125	39.41%	3202	40.38%	1603	20.21%	
**Coronary heart disease**							*<0.001*
No	3122	39.6%	3180	40.3%	1580	20.0%	
Yes	3	6.3%	22	45.8%	23	47.9%	
**Total**	2265	38.84%	2372	40.68%	1194	20.48%	

## Discussion

Previous studies have shown variable estimates of obesity and overweight in KSA, which may stem from non-representative samples or reliance on self-reported anthropometric data [16,17]. Using data from the 2019 KSAWHS survey, this study provides nationally representative estimates of obesity, as well as associated risk factors and comorbidities. These findings offer a baseline for the 2024 national survey planned by the Saudi MOH.

Our findings are consistent with prior studies. Althumiri et al. reported an obesity prevalence of 24.7% using self-reported BMI [16]. Similarly, a Makkah region survey estimated overweight and obesity prevalence at 32.8% and 23%, respectively [17]. WHO data from 2016 reported higher national prevalence rates of overweight (67.5%) and obesity (29.5%) [15]. Variability in these estimates may reflect methodological differences. However, our results suggest a lower prevalence of obesity and overweight in KSA compared to other GCC countries, such as the UAE, where rates are 43.0% and 32.3%, respectively [13].

The decline in obesity prevalence in KSA coincides with national efforts under Vision 2030 to promote physical activity, healthy diets, and preventive health [22]. Initiatives include mandatory calorie labeling on food, increased taxes on sugary drinks [16], and public awareness campaigns [23]. Programs like the ‘Quality of Life Program’ and the ‘Obesity Control Program’ further emphasize this focus [24]. These policy efforts, coupled with heightened community awareness, may have contributed to a decreasing trend, though further investigation is required.

This study also identified regional and gender-specific disparities in obesity prevalence. Consistent with earlier findings, obesity was more prevalent in females, while overweight was more common in males [17,25–28]. Regional variations in prevalence, reported in both this study and earlier surveys [16,29], may reflect environmental, genetic, and behavioral factors and highlight the need for region-specific interventions.

Behavioral and dietary patterns play a critical role in obesity. While prior studies show an inverse relationship between fruit and vegetable intake and obesity risk [30–32], this study found no significant association, likely due to reliance on self-reported dietary data subject to recall bias.

Obesity is strongly linked to comorbidities such as T2DM, hypertension, dyslipidemia, and cardiovascular diseases, consistent with prior evidence [33–35]. In this study, these conditions were more prevalent among obese and overweight individuals, underscoring the broader health implications of excess weight.

## Conclusion

This study provides important data that comprises nationally representative sample and rigorous survey design; however, it is limited by reliance on outdated census data (2010), self-reported measures prone to recall bias, and the inability to assess genetic factors or establish causality. With obesity and overweight prevalence at 20.3% and 38.7%, respectively, these findings provide a critical baseline for evaluating trends in KSA. The upcoming 2024 national survey will assess the impact of current policies, guide resource allocation, and inform targeted interventions, underscoring the urgent need to address the significant health and economic burden posed by obesity and overweight.

## Supplementary Material

Paper Context_Obesity.docx
