# Supplementary material for: Comorbidities of overweight and obesity associated risk factor in Saudi Arabia: a population-based analysis
Source: Glob Health Action. 2025 Apr 9;18(1):2477387. doi: 10.1080/16549716.2025.2477387 (PMC11983525; doi:10.1080/16549716.2025.2477387)
Supplement: Paper Context_Obesity.docx [file ZGHA_A_2477387_SM5152.docx]

**The Prevalence, Behavioral Risk Factors, and Comorbidities of Overweight and Obesity in Saudi Arabia: A Population-based Analysis**

**Study Highlights**

- **Main Findings:**
- The prevalence of obesity and overweight in KSA was 20.3% and 38.7%, respectively, indicating a substantial public health burden.
- Obesity was significantly associated with metabolic, cardiovascular, and psychological disorders.
- Key behavioral risk factors included physical inactivity, poor dietary habits, and smoking.
- **Added Knowledge:**
- Provides updated, nationally representative data on obesity trends and risk factors in KSA.
- Highlights the role of lifestyle behaviors in obesity prevalence and associated comorbidities.
- Identifies critical targets for intervention, emphasizing the need for evidence-based health strategies.
- **Global Health Impact for Policy and Action:**
- Supports the development of targeted public health policies to combat obesity-related diseases.
- Aligns with global efforts to reduce non-communicable diseases (NCDs) through preventive strategies.
- Reinforces the need for multi-sectoral approaches, including healthcare, education, and urban planning, to promote healthier lifestyles and reduce obesity rates.
